# Psychoacoustic Tinnitus Loudness and Tinnitus-Related Distress Show Different Associations with Oscillatory Brain Activity

**DOI:** 10.1371/journal.pone.0053180

**Published:** 2013-01-10

**Authors:** Tobias Balkenhol, Elisabeth Wallhäusser-Franke, Wolfgang Delb

**Affiliations:** Department of Phoniatrics and Audiology, Medical Faculty Mannheim, Heidelberg University, Mannheim, Germany; University of Regensburg, Germany

## Abstract

**Background:**

The phantom auditory perception of subjective tinnitus is associated with aberrant brain activity as evidenced by magneto- and electroencephalographic studies. We tested the hypotheses (1) that psychoacoustically measured tinnitus loudness is related to gamma oscillatory band power, and (2) that tinnitus loudness and tinnitus-related distress are related to distinct brain activity patterns as suggested by the distinction between loudness and distress experienced by tinnitus patients. Furthermore, we explored (3) how hearing impairment, minimum masking level, and (4) psychological comorbidities are related to spontaneous oscillatory brain activity in tinnitus patients.

**Methods and Findings:**

Resting state oscillatory brain activity recorded electroencephalographically from 46 male tinnitus patients showed a positive correlation between gamma band oscillations and psychoacoustic tinnitus loudness determined with the reconstructed tinnitus sound, but not with the other psychoacoustic loudness measures that were used. Tinnitus-related distress did also correlate with delta band activity, but at electrode positions different from those associated with tinnitus loudness. Furthermore, highly distressed tinnitus patients exhibited a higher level of theta band activity. Moreover, mean hearing loss between 0.125 kHz and 16 kHz was associated with a decrease in gamma activity, whereas minimum masking levels correlated positively with delta band power. In contrast, psychological comorbidities did not express significant correlations with oscillatory brain activity.

**Conclusion:**

Different clinically relevant tinnitus characteristics show distinctive associations with spontaneous brain oscillatory power. Results support hypothesis (1), but exclusively for the tinnitus loudness derived from matching to the reconstructed tinnitus sound. This suggests to preferably use the reconstructed tinnitus spectrum to determine psychoacoustic tinnitus loudness. Results also support hypothesis (2). Moreover, hearing loss and minimum masking level correlate with oscillatory power in distinctive frequency bands. The lack of an association between psychological comorbidities and oscillatory power may be attributed to the overall low level of mental health problems in the present sample.

## Introduction

Tinnitus is an auditory percept that does not originate from a physical sound source but is generated within the auditory system. Therefore, a subjective tinnitus is heard only by the affected individual. Cochlear hearing impairment is seen as a permissive if not a necessary condition for tinnitus [1]–[3]. As hearing impairments become more common with advancing age, it is not surprising that the prevalence of tinnitus increases with age [3], [4]. Although tolerated well by many, tinnitus may be the cause for substantial deterioration of life quality [5]. Concerning the impact of tinnitus on an individual, a perceptive component reflected by the subjectively perceived tinnitus loudness and an affective component reflected by the amount of tinnitus-related distress are distinguished [6], [7]. In particular, severely distressing tinnitus tends to be associated with increased levels of depressivity, anxiety, and somatic symptom severity [6], [8], [9].

As a consciously experienced, often continuous, and prominent signal tinnitus should be represented in the spontaneous activation pattern of the cortex. In line with this assumption, magnetoencephalographic (MEG) studies showed that the presence of tinnitus is associated with increased gamma band activity in the auditory cortex (AC) [10]–[12]. This finding is corroborated by electroencephalographic (EEG) studies that demonstrate the emergence of elevated gamma activity in persons who experience acute tinnitus [13]. Furthermore, gamma band activity in the AC shows some correlation with tinnitus intensity [14], and enhanced gamma activity is localized contralateral to the tinnitus ear in individuals with unilateral tinnitus (MEG: [12], EEG: [15]). Synchronization of fast oscillatory responses in the beta and gamma range is increased during demanding tasks that involve cooperation of widespread cortical regions. This is seen in a variety of cognitive tasks that require routing of signals across distributed cortical networks, perceptual grouping, attention-dependent stimulus selection, sensory-motor integration, working memory, and perceptual awareness [16]. Both synchronization and strength of neuronal oscillations in the gamma frequency range influence the amount and speed of information transfer [17].

At the same time alpha oscillatory activity is decreased in subjects with tinnitus compared to non-tinnitus controls [11], [12], [18]. Sensory systems exhibit pronounced alpha-like oscillatory activity during resting conditions. Therefore, low levels of alpha activity are thought to reflect a state of excitation while high levels are linked to reduced excitatory drive [19]. Weisz and coworkers [2], [20] proposed that the dominant alpha activity at rest is functionally related to ongoing inhibitory activity that prevents spontaneous synchronization of cell assemblies. In line with this interpretation, auditory alpha activity, which also is referred to as tau activity [21], desynchronizes during presentation of auditory stimuli [20]. Thus, reduced alpha oscillatory power as seen in tinnitus patients suggest that tinnitus is associated with loss of cortical inhibition, a notion that is corroborated by findings of a down regulation of inhibition in deafferented regions of the AC in animal models of tinnitus [22], and the finding that functional deafferentation of central auditory areas by hearing loss leads to a significant reduction of alpha power in humans [23].

In the clinical setting a variety of audiological tinnitus characteristics are measured of which tinnitus loudness and tinnitus maskability are particularly important for the patient and the therapist. Tinnitus loudness is determined by different matching procedures, but the results of these measurements are not always satisfactory because they do not necessarily represent the patient's subjectively perceived tinnitus loudness. Minimum masking level on the other hand describes the minimal noise level that is necessary to eliminate the tinnitus perception, and represents a patient's ability to effectively use environmental sound to control the tinnitus perception. While there have been reports on correlates of tinnitus loudness in oscillatory brain activity, electrophysiological correlates of tinnitus maskability and the underlying mechanism remain unclear. In our study we set out to test the following hypotheses on tinnitus and spontaneous oscillatory brain activity:

Hypothesis (1): Loudness of the tinnitus sound correlates with gamma band oscillatory power during absence of external auditory stimulation. Conventionally, tinnitus loudness is measured by a variety of audiological matching procedures (see [24] for a review) or by subjective rating scales [25]. Both methods have limitations. Whereas loudness estimates derived by subjective rating scales are likely to be influenced by the distress attributed to the tinnitus, matching to pure tones at the tinnitus frequency or at 1 kHz might underestimate its loudness, since even if patients describe their tinnitus as extremely loud, measurements are usually found to be only a few dB above threshold [24]. Therefore we developed a new method to reconstruct the tinnitus sound, resulting in sounds that closely matched the individual tinnitus percept of a patient. We hypothesized, that tinnitus loudness estimates derived by comparison to sound synthesized in that way show a better correlation with brain activity than tinnitus loudness estimates derived by comparison to pure tones that are less similar to the tinnitus.

While many of the publications including those cited above compare tinnitus subjects with non-tinnitus subjects, the relation between the subjectively perceived tinnitus loudness and brain oscillatory activity has only been addressed by van der Loo and coworkers [14], and up to now there is no report on the association of tinnitus loudness determined by matching with an external auditory stimulus and oscillatory brain activity.

Hypothesis (2): Tinnitus loudness and tinnitus-related distress are associated with distinct spontaneous brain oscillations. From patient reports it is evident that tinnitus loudness and tinnitus-related distress are distinct characteristics of the tinnitus [6], [26], therefore we hypothesized that tinnitus loudness and tinnitus-related distress correlate with distinct aspects of brain activity. Estimates of tinnitus-related distress were derived from a self-report questionnaire.

In the exploratory part of the present study we focused on the following aspects:

(3) We explored the association between oscillatory band power and hearing loss as well as minimum masking level, which are both highly relevant for patients. The relevance of MML has been outlined above and hearing impairment is seen as a permissive, although not sufficient condition for the establishment of tinnitus [1]–[3]. According to the model originally proposed by Llinas et al. [10] hearing impairment should be related to oscillatory brain activity. To the best of our knowledge, this is the first study that addresses this aspect.

(4) Finally, we explored how psychological comorbidities that often accompany tinnitus [6], and that are known to influence oscillatory brain activity, have distinct influence on oscillatory brain activity in tinnitus patients. Even though the relation between tinnitus-related distress and oscillatory brain activity has been addressed repeatedly [11], [27], [28], comorbidities such as depressivity and anxiety [6] have not been taken into account.

Since gender differences and oversensitivity to external sounds (hyperacusis) might influence resting state EEG power distribution [29], tinnitus and non-tinnitus participants were restricted to males with normal sound sensitivity.

## Methods

The present study was approved by the ethics committee of the Medical Faculty Mannheim (Ethikkommission II) of Heidelberg University according to the principles expressed in the Declaration of Helsinki. Subjects were acquired by newspaper advertisements and consecutively enrolled in the study. All subjects of the patient and the control group were informed about aim and scope of the study and gave written consent. All participants were males and right handed.

### Tinnitus patient group

**Figure 1:**
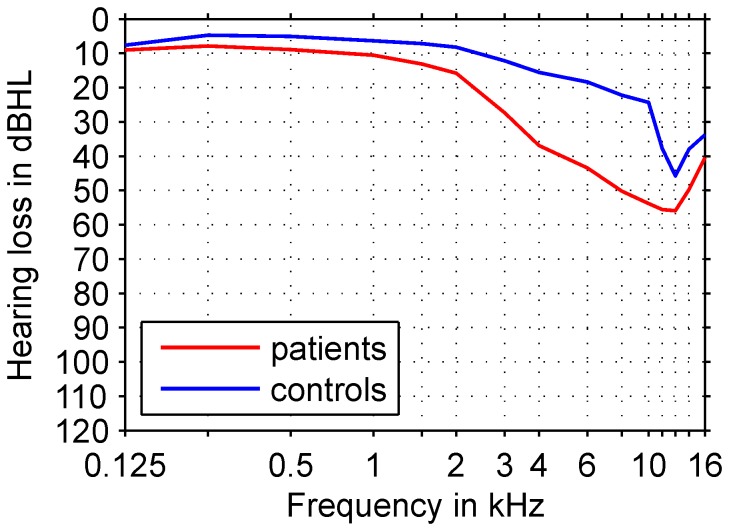
Averaged audiograms of the patient and control groups. Hearing ability was determined between 0.125 kHz and 16 kHz. Group means are shown. Tinnitus patients exhibit more pronounced hearing loss than controls above 2 kHz. Note that the controls as a group exhibit noticeable hearing impairment above 10 kHz.

Mean age of the 46 tinnitus patients included in the study was 54.8 years (range 22 to 68 years) and it did not differ from that of the control group (ANOVA: 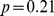). Tinnitus was present bilaterally in 27 and unilaterally in 19 (left: 12; right: 7). Pure tone tinnitus was experienced by 40 participants while 6 had noise-like tinnitus. Mean hearing level (MHL) in the frequency range from 0.125 kHz to 16 kHz was 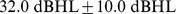 (Fig. 1). Only 4 subjects had a highly distressing tinnitus according to the Tinnitus Questionnaire (TQ Hallam et al. [30], German version [31]) with a main score above 47. Average uncomfortable loudness thresholds (UCL) between 0.125 kHz and 10 kHz of all tinnitus patients in the study were normal with 85 dBHL or above.

### Control group

None of the 10 participants included in the control group had a history of tinnitus or any other type of ear-related pathology, and all had scores of 60 or below in any of the Symptom Ckecklist-90-R (SCL-90-R) subscales indicating unproblematic psychological conditions. Mean age was 50.4 years (range 25 to 62 years), and hearing loss between 0.125 kHz and 16 kHz averaged to 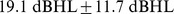 (Fig. 1, Table 1).

**Table 1:** Participant characteristics.

Parameter	Tinnitus group	Control group	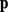 -value
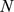	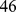	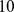	-
Age (years)	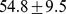	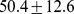	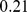
UCL (dBHL)	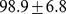	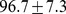	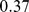
MHL (dBHL)	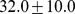	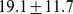	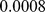
GSI	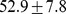	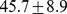	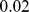
PSDI	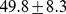	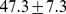	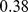
PST	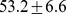	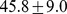	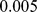
DEP	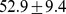	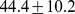	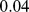
SOM	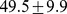	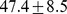	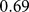
ANX	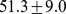	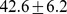	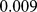
TinDur (years)	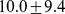	-	-
TinDis	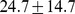	-	-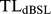 (dBSL)	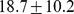	-	-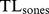 (sones)	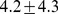	-	-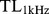 (dBHL)	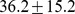	-	-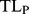 (dBHL)	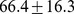	-	-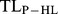 (dBSL)	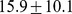	-	-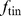 (kHz)	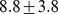	-	-
MML (dBHL)	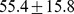	-	-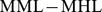 (dBSL)	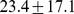	-	-
RTS	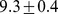	-	-

Group means and standard deviations are reported. Auditory measures: mean hearing loss (MHL, left and right ear averaged for the frequency range 0.125 kHz to 16 kHz); mean threshold of uncomfortable loudness (UCL, left and right ear averaged for the frequency range 0.125 kHz to 10 kHz). Psychological measures derived from the SCL-90-R: global severity index (GSI), positive symptom total (PST), positive symptom distress index (PSDI), depressiveness subscale (DEP), somatization subscale (SOM), anxiety subscale (ANX). Tinnitus characteristics: tinnitus duration (TinDur), tinnitus-related distress (TinDis) derived from the Tinnitus Questionnaire (scores 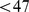: low tinnitus-related distress, scores 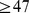: high tinnitus-related distress). Tinnitus loudness measures (TL, see section “Psychoacoustic measurements” and Table 2); frequency of the major peak in tinnitus spectrum (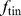); minimum masking level when masking with white noise (MML) as well as minimum masking level above mean hearing threshold (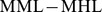); rating of similarity of reconstructed tinnitus sound to own tinnitus (RTS, 0: no match, 10: perfect match).

### Psychoacoustic measurements

Thresholds were measured in 1 dB steps with pure tones at the standard frequencies of the audiogram (range from 0.125 kHz to 10 kHz) and in addition at 11.2 kHz, 12.5 kHz, 14 kHz, and 16 kHz (audiometer: Auritec AT900; headphones: Sennheiser HDA200). Mean hearing loss (MHL) was calculated by averaging across all frequencies 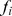.

Uncomfortable loudness thresholds (UCL) were recorded at the standard frequencies. For this purpose the sound pressure level of each pure tone was presented at hearing threshold and its level was increased continuously until the sound became uncomfortable. Subjects indicated UCL by pressing a button.

Minimum masking levels (MML) were determined with white noise at the tinnitus ear. In cases of bilateral tinnitus MML was determined for each ear. White noise was presented at hearing threshold and increased in 1 dB step sizes until it masked a subject's tinnitus, which the subject indicated by pressing a button.

#### Tinnitus reconstruction

Tinnitus reconstruction was based on psychoacoustic tinnitus spectra as described earlier [32] and expanded to a novel heuristic, easy to handle method. Reconstructions were performed with 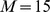 pure tones at the standard frequencies and with the additional high frequencies 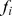 of the audiogram (see above). Pure tones were presented to the tinnitus ear or to the ear with less hearing loss in cases of bilateral tinnitus. First, a given pure tone was adjusted to the perceived tinnitus loudness, then the patient rated its contribution to his tinnitus on a numeric rating scale (0: no contribution, 10: perfect match). This was repeated three times and ratings were averaged for each frequency 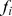. Thereupon average scores were processed with a custom MATLAB script (The Mathworks, Natick, Massachusetts, USA) to synthesize the tinnitus sound which was played back monaurally at a sampling rate of 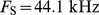. Pure tone tinnitus 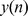 was synthesized by processing the averaged scores 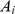 as follows:
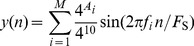
(1)During play back of the generated sound to the patients via headphone (Sennheiser HDA200), loudness of each frequency component (parameter 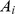) was fine-tuned by the examiner on a graphical user interface. The procedure was stopped if no further improvements of the matching score were achieved.

Amplitude modulated tinnitus was approximated by
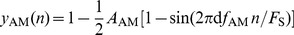
(2)with the parameters 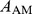 and 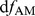 representing modulation amplitude and frequency. If tinnitus contained a noise component the corresponding tinnitus spectrum was reconstructed in a last step by:
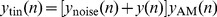
(3)For adjusting 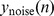, white noise was band-pass filtered and the cutoff frequencies were selected according to the noise spectrum in a patient's tinnitus. Note that the reconstructed tinnitus covered all frequency components up to 16 kHz.

Averaged across all tinnitus participants, similarity of the reconstructed tinnitus sound to a patient's own tinnitus reached an average similarity index of 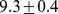 when rated on a numeric rating scale (0: no contribution, 10: perfect match), indicating a very good fit of the reconstructed tinnitus sound. Averaged across all tinnitus participants, the major peak of the reconstructed tinnitus spectrum was located at 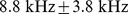 (Table 1 for details).

#### Tinnitus loudness

Overall five different tinnitus loudness estimates were determined (Table 2). The first tinnitus estimate was obtained in a monaural matching procedure using the reconstructed tinnitus sound as described above and MHL was subtracted from the whole tinnitus spectrum. The largest peak in the spectrum was defined as the tinnitus loudness estimate 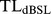. In addition, tinnitus loudness was calculated in sone as
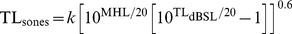
(4)with 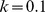 (see [24], [33]).

**Table 2:** Psychoacoustic measures for tinnitus loudness.

Loudness measure	Stimulus for matching procedure	Measure calculated as
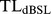	reconstructed tinnitus sound	MHL is subtracted from the level of the major peak in the psychoacoustic tinnitus spectrum after loudness matching
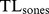	reconstructed tinnitus sound	see equation (4)
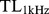	sine tone at 1 kHz	-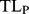	sine tone at the major peak of the psychoacoustic tinnitus spectrum	-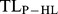	sine tone at the major peak of the psychoacoustic tinnitus spectrum	hearing loss at the major peak of tinnitus spectrum is subtracted from 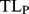

Overview on the types of stimuli generated for psychoacoustic tinnitus loudness matching (see section “Psychoacoustic measurements”).

Third, in order to account for recruitment phenomena [33], a 1 kHz pure tone was presented via headphone and its loudness was adjusted in 1 dB steps until it was perceived by the patient as loud as his own tinnitus (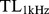). A further loudness measure 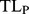 was generated by matching the loudness of the pure tone which corresponded to the major peak of the tinnitus spectrum to the tinnitus loudness experienced by the patient. Finally, the loudness measure 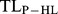 was obtained by subtracting hearing loss at the major peak of the tinnitus spectrum from 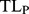.

### Tinnitus-related distress and psychometric testing

Tinnitus-related distress was evaluated with the Tinnitus Questionnaire (TQ Hallam et al. [30], German version [31]). This 52 item questionnaire yields a sum-score between 0 to 84 and estimates separate subscores for emotional distress, cognitive distress, intrusiveness, auditory perceptual difficulties, sleep disturbance, and somatic complaints. Sum-scores below 47 indicate low to moderate tinnitus-related distress, whereas values of 47 and above indicate high to very high tinnitus-related distress.

In addition, the German version of the Symptom Checklist-90-R (SCL-90-R [34], [35]) was completed by all participants. The SCL-90-R contains subscales for somatization, obsessive-compulsive behavior, interpersonal sensitivity, depression, anxiety, hostility, phobic anxiety, paranoid ideation, and psychoticism. Beyond that, the following global scores were derived: The global severity index (GSI) sets the intensity of perceived distress in reference to all items of the SCL-90-R and is the best single predictor for the current level or depth of mental distress. The positive symptom total (PST) score is a measure for the quantity of items indicating distress. The positive symptom distress index (PSDI) reflects the average level of distress reported for individual symptoms and it is interpreted as a measure of symptom intensity. Combination of the subscores GSI, PSDI, and PST yields a general psychological distress estimate (GPD).

### EEG recording

EEG recordings took place in a dimly lit sound booth shielded against electromagnetic interference (EMI) and connected with the recording room via a glass window. Participants were seated comfortably with uncrossed arms and legs in an armchair that had a head-rest. They were instructed to relax and to avoid any movements.

Eyes were closed during EEG recording, and analysis was confined to resting EEG recorded for 120 s. A cap (g.GAMMAcap, g.tec Medical Engineering GmbH, Austria) with 22 sintered Ag/AgCl surface electrodes was placed at the standard positions of the extended 10–20 system (Fp1, Fp2, F7, F3, F1, Fz, F2, F4, F8, T7, C3, Cz, C4, T8, P7, P3, Pz, POz, P4, P8, O1, O2) and referenced to linked ear lobes. The electrooculogram (EOG) was monitored with 4 sintered Ag/AgCl surface electrodes (LO1, LO2, IO1, IO2). Impedances were checked to be below 5 kOhm and the sampling rate was set to 512 Hz. EEG signals were acquired by two cascaded 24 bit biosignal amplification units (g.USBamp, g.tec Medical Engineering GmbH, Austria). EEGs were inspected for indicators of sleep such as spindles, enhanced theta oscillations or a slowed alpha rhythm, and only subjects who stayed awake were included.

### Data preprocessing and editing

EEG data were pre-processed and analyzed offline with MATLAB. Slow fluctuations were removed by local linear regression (see http://chronux.org/ for details). Length of the moving window and step size were set to 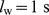 and 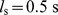, respectively. Artifacts at 50 Hz and multiples due to power line interferences were removed by adaptive filter techniques using a separate adaptive filter with two filter coefficients for each interference frequency [36].

Episodic artifacts including muscle artifacts, eye blinks, teeth clenching, or body movement were removed by visual inspection using the MATLAB scripts of EEGLAB [37]. EOG artifacts were removed automatically with a custom MATLAB script by applying the following steps: Low-pass filtering of EOG channels with 5 Hz cutoff frequency, decomposing EEG and EOG signals into independent components with a second order blind identification algorithm [37], [38], selection of the EOG components according to their correlation with the recorded EOG channels, high-pass filtering of the selected EOG components with 5 Hz cutoff frequency to remove identified EOG artifacts, and reconstruction of the EEG signal. Subsequent high-pass filtering of the EOG components ensured that automatic artifact removal was restricted to frequencies below 5 Hz where EOG artifacts were expected. After visual artifact removal mean length of the recording was 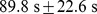.

Power spectral estimation and analysis was done with a multi-taper method (see http://chronux.org/ and [39], [40] for details) that tapers the time series by an optimal set of orthogonal tapers (Slepian functions) and applies a Fourier transformation. With the chosen time-bandwidth-product 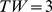 and the relation 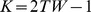 a total number of 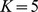 tapers were used for power spectral estimation. Mean power spectra were determined by averaging the log-transformed power density spectra of all scalp electrodes for each subject and calculated separately for delta (0.5 Hz to 3 Hz), theta (4 Hz to 7 Hz), alpha (8 Hz to 13 Hz), beta (14 Hz to 30 Hz), and gamma (31 Hz to 64 Hz) band frequencies. Frequencies near the power line artifacts 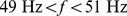 were excluded before averaging results in the gamma frequency range. Because of the relatively low number of 22 electrodes which results in low localization precision [41] we did not apply source localization algorithms.

### Statistics

Spearman's rank correlation coefficient 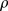 was computed for spectral power in the different frequency bands and the psychoacoustic and psychometric factors following a custom MATLAB script [42]. A false discovery rate (FDR) correction was applied to correct for multiple comparisons [43].

## Results

### Power spectra

An initial ANOVA did not show significant differences for any frequency band between the tinnitus and the control group when averaging power across all 22 electrodes (delta 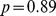, theta 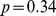, alpha 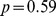, beta 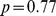, gamma 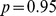), whereas more detailed correlation analyses revealed significant interactions between tinnitus loudness, tinnitus-related distress, hearing loss and oscillatory band power depending on type of tinnitus loudness measure, oscillation frequency, and control for confounding factors.

### Correlation analyses

#### Mean hearing loss (MHL)

MHL did not correlate significantly with oscillatory power averaged over all electrodes in any frequency band, no matter whether controlled for age, general psychological distress (GPD), and tinnitus loudness in dBSL (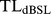) or not. When performing the same analysis with tinnitus loudness estimates in the sone scale (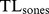), however, a significant decrease of gamma band power with increasing MHL became apparent (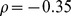, 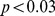). Restricting the analysis to patients with pure tone tinnitus and controlling for age, GPD, and 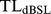, a weakly significant correlation between MHL and alpha band power (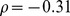, 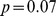) became apparent, while correlations of MHL and band power averaged over all electrodes in this group reached significance in the alpha, beta, and gamma band when using the tinnitus loudness estimate 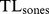 (Table 3). For controls, the correlation between MHL and theta band power reached significance (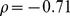, 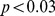).

**Table 3:** Partial correlation of band power averaged over all electrodes with audiological parameters for the subgroup with pure tone tinnitus.

		delta		theta		alpha		beta		gamma	
Parameter	controlled for (partial correlation)	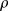	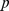	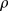	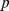	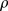	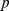	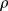	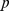	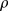	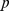
MHL	age, GPD, 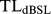	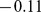	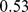	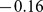	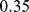	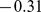	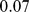	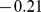	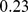	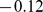	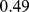
MHL	age, GPD, 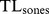	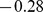	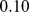	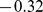	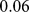	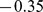	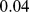	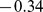	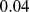	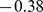	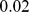
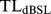	age, GPD, MHL	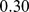	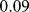	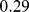	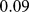	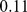	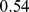	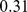	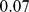	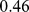	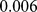
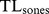	age, GPD, MHL	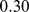	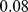	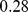	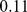	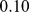	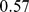	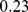	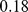	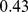	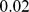
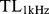	age, GPD, MHL	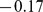	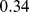	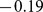	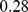	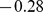	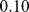	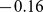	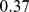	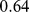	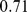
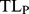	age, GPD, MHL	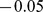	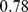	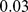	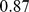	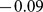	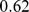	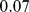	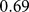	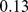	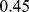
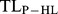	age, GPD, MHL	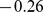	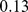	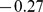	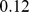	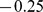	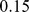	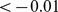	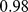	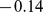	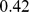
MML	age, GPD, MHL	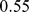	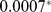	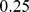	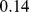	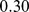	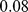	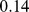	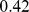	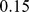	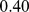
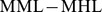	age, GPD, MHL	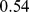	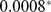	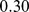	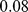	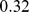	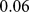	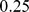	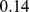	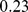	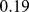

Correlation coefficients (Spearman's 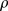) and corresponding significance levels (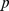) for tinnitus loudness (TL), minimum masking level (MML), mean hearing loss (MHL) with oscillatory band power in the delta to gamma range are reported. MHL: mean hearing loss averaged for left and right ears and for the frequencies between 0.125 kHz and 16 kHz; TL: tinnitus loudness measures (see section “Psychoacoustic measurements” and Table 2); MML: minimum masking level with white noise; 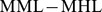: minimum masking level with white noise above mean hearing threshold. Significant correlations (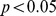) are indicated by bold letters. Correlations which remained significant after FDR correction (FDR 0.05) are denoted by 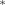 at the corresponding 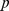-value.

#### Tinnitus loudness

The correlations of all tinnitus loudness measures (Table 2) with tinnitus-related distress, MHL, and MML are summarized in Table 4. Statistical significance of correlations between these factors depended on the type of loudness measure that was used.

**Table 4:** Correlations of tinnitus loudness and distress with auditory parameters.

	TinDis		MHL		MML		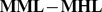	
Parameter	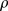	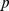	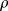	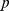	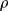	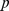	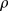	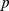
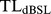 (dBSL)	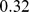	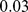	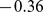	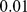	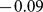	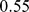	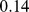	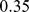
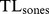 (sones)	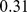	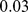	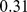	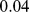	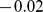	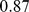	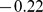	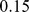
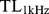 (dBHL)	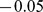	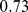	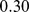	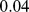	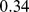	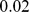	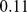	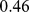
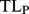 (dBHL)	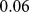	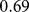	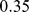	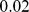	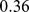	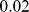	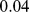	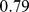
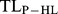 (dBSL)	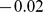	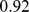	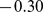	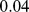	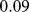	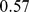	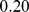	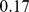

For the patient group, correlation coefficients (Spearman's 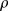) and corresponding significance levels (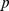) for each of the five different measures for tinnitus loudness (TL, see section “Psychoacoustic measurements” and Table 2) with tinnitus-related distress (TinDis), mean hearing loss (MHL), minimum masking level (MML), as well as minimum masking level above mean hearing threshold (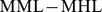). Mean hearing loss (MHL) was averaged for left and right ears and for the frequency range between 0.125 kHz and 16 kHz. Minimum masking level (MML) was measured with white noise. Significant correlations (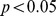) are indicated by bold letters. Correlations did not remain significant after FDR correction (FDR 0.05).

Similarly, statistical significance of correlations between tinnitus loudness and oscillatory brain activity depended on the type of tinnitus loudness that was used (Table 3 and 5). Tinnitus loudness 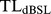 showed a weakly significant correlation with band power averaged over all electrodes in the gamma (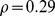, 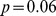) band. Significance of this correlation improved (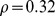, 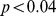) when controlling for age, GPD, and MHL, and it improved even more when controlling for these factors and tinnitus-related distress in addition (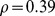, 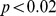, Table 5).

**Table 5:** Correlation of band power averaged over all electrodes with audiological and psychological parameters of the patient group.

		delta		theta		alpha		beta		gamma	
Parameter	controlled for (partial correlation)	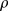	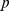	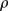	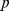	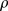	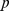	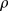	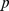	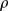	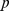
Age	-	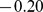	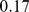	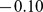	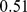	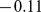	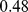	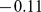	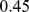	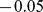	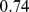
MHL	-	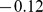	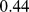	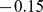	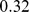	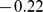	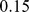	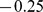	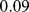	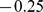	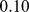
	age, GPD, 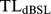	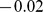	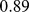	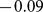	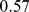	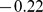	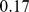	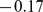	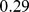	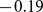	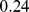
	age, GPD, 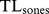	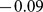	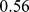	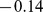	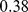	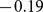	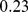	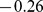	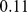	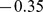	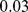
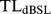	-	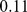	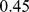	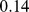	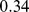	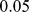	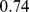	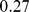	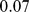	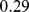	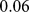
	age, GPD, MHL	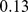	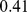	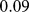	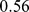	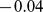	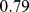	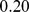	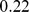	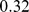	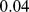
	age, GPD, MHL, TinDis	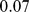	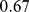	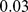	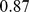	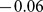	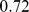	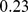	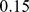	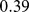	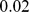
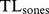	-	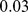	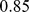	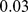	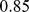	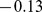	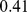	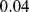	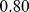	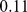	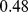
	age, GPD, MHL	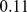	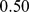	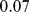	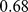	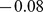	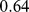	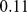	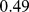	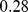	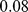
	age, GPD, MHL, TinDis	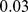	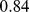	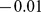	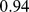	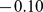	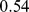	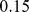	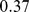	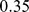	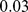
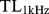	-	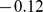	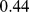	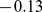	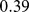	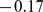	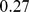	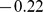	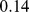	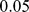	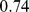
	age, GPD, MHL	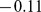	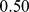	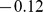	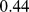	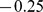	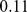	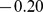	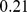	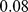	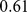
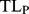	-	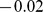	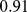	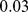	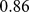	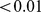	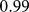	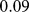	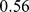	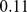	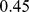
	age, GPD, MHL	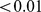	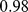	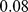	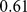	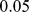	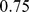	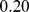	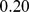	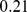	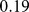
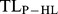	-	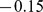	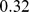	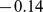	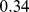	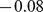	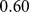	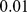	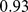	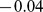	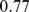
	age, GPD, MHL	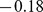	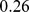	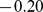	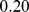	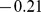	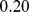	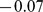	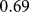	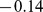	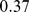
MML	-	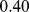	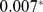	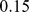	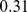	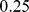	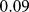	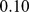	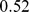	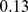	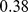
	age, GPD, MHL	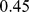	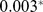	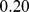	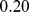	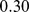	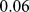	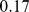	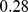	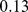	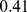
	age, GPD, MHL, 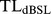	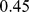	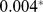	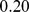	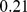	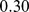	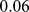	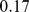	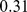	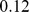	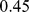
	age, GPD, MHL, TinDis	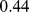	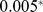	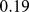	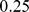	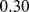	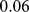	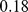	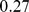	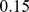	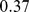
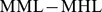	-	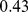	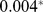	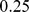	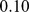	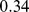	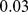	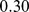	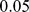	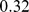	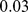
	age, GPD, MHL	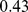	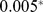	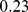	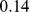	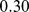	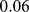	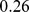	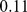	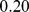	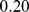
TinDur	-	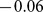	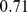	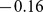	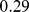	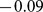	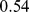	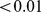	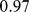	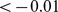	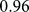
TinDis	-	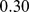	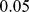	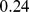	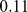	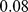	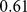	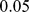	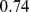	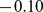	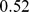
	GPD	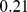	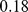	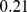	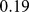	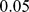	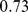	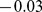	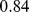	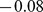	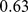
	DEP	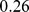	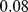	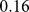	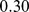	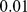	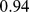	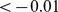	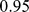	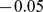	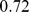
	SOM	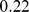	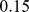	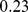	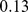	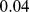	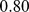	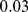	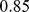	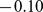	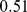
	ANX	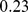	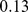	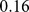	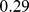	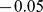	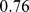	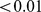	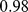	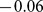	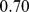
	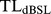	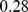	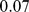	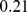	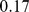	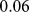	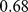	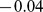	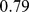	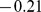	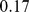
GSI	-	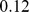	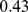	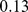	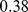	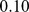	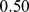	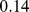	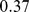	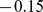	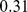
PSDI	-	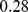	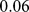	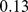	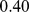	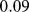	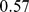	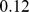	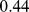	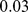	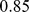
PST	-	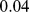	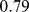	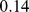	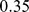	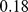	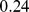	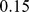	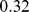	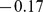	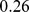
DEP	-	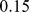	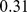	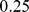	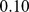	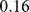	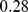	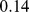	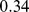	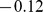	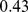
SOM	-	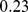	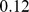	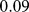	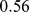	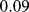	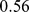	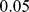	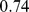	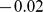	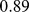
ANX	-	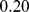	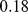	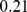	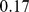	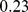	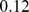	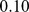	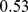	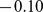	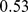

Correlation coefficients (Spearman's 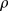) and corresponding significance levels (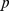) between tinnitus characteristics, minimum masking level (MML), mean hearing loss (MHL), tinnitus-related distress (TinDis), psychometric testing scores and oscillatory band power in the delta to gamma range are reported for the whole tinnitus group. MHL: mean hearing loss averaged for left and right ears between 0.125 kHz and 16 kHz; TL: tinnitus loudness measures (see section “Psychoacoustic measurements” and Table 2); MML: minimum masking level with white noise; 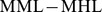: minimum masking level with white noise above mean hearing threshold; TinDur: tinnitus duration in years; TinDis: tinnitus-related distress; DEP: depression subscale of the SCL-90-R; SOM: somatization subscale of the SCL-90-R; ANX: anxiety subscale of the SCL-90-R. Significant correlations (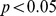) are indicated by bold letters. Correlations which remained significant after FDR correction (FDR 0.05) are denoted by 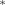 at the corresponding 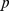-value.

**Figure 2:**
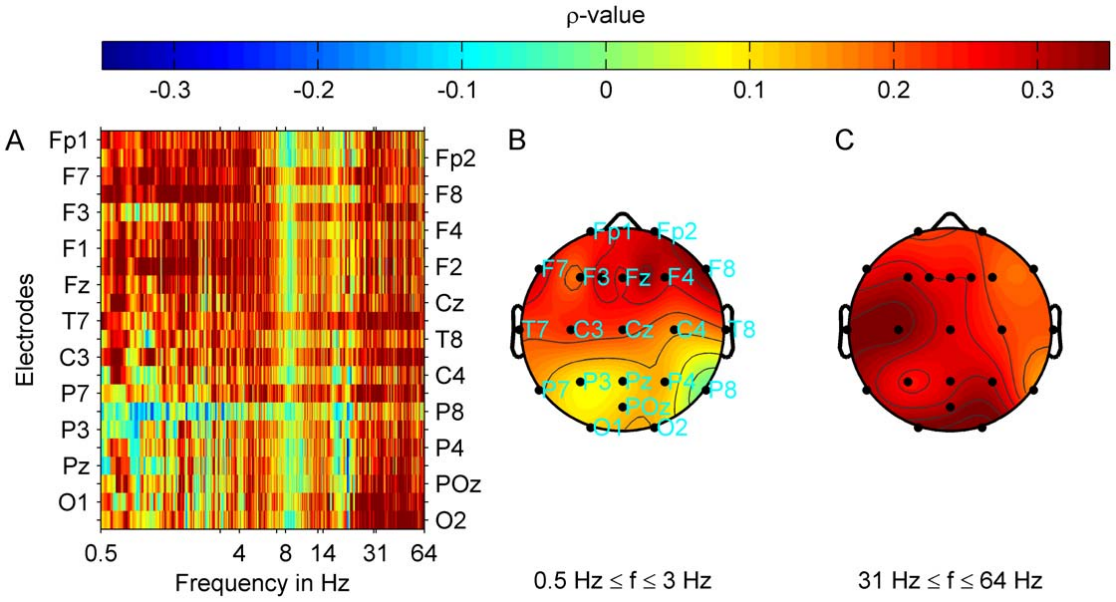
Spatio-spectral distribution of correlation strength between tinnitus loudness and oscillatory band power for the subgroup with pure tone tinnitus. Group averages are shown. Power spectra were interpolated with a resolution of 40 points per 1 Hz. Tinnitus loudness was determined by adjusting the contribution of each frequency component and the loudness of such a reconstructed tinnitus spectrum to the perceived tinnitus. Correlations were controlled for age, global psychological distress (GPD), and mean hearing loss (MHL) between 0.125 kHz and 16 kHz. (A) Correlation strength (Spearman's 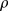) at each electrode and frequency point is shown. Plots (B) and (C) show correlation maps corresponding to (A) with averaged correlation strength (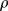) topographies for the tinnitus loudness 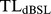 and delta (B) or gamma (C) oscillatory power. Correlation strength for delta band power and tinnitus loudness was highest in the frontal half of the brain and lowest at posterior locations. For the correlation between gamma band power and tinnitus loudness the distribution of correlation strength across electrode positions was more uniform. Highest correlation strength was reached at the left temporal and right occipital electrode positions. After FDR correction (FDR 0.05: 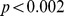) correlations remained significant at all electrode positions except for T8 and P8 locations for the gamma band, whereas significant correlations in the delta band were attained at the fronto-central locations Fp2, F1, Fz, F2, F4, F8, C3, Cz, and at P7.

When restricting the analysis to patients with pure tone tinnitus (Table 3), the partial correlation between band power averaged over all electrodes and 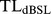 controlled for age, GPD, and MHL became highly significant for the gamma (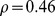, 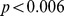) band. Fig. 2 shows that correlation strength across the 22 electrode positions was more uniform for the correlation with gamma (Fig. 2C) than with delta band power (Fig. 2B). These correlations remained significant after correction for multiple comparison (FDR 0.05: 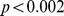) in the delta range at the fronto-central electrode positions Fp2, F1, Fz, F2, F4, F8, C3, Cz, and P7, and in the gamma range at all but the T8 and P8 electrode positions. Similar results were seen for delta and gamma band when performing a partial correlation between tinnitus loudness in sone (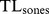) and band power controlled for age, GPD, and MHL in patients with pure tone tinnitus (Table 3).

**Figure 3:**
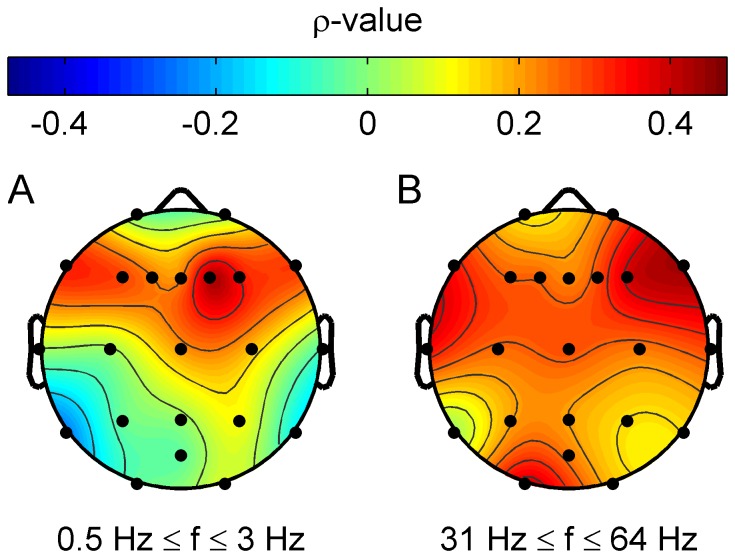
Correlation strength between tinnitus loudness and oscillatory band power for the subgroup with unilateral pure tone tinnitus. Group averages are shown. Electrode positions of left and right hemisphere were interchanged for right-sided tinnitus. Left ear in the plots is the tinnitus ear. Power spectra were interpolated with a resolution of 40 points per 1 Hz. Tinnitus loudness was determined by matching the contribution of each frequency component and the loudness of such a reconstructed tinnitus spectrum to the perceived tinnitus. Correlations with oscillatory band power were controlled for age, global psychological distress (GPD), and mean hearing loss (MHL) between 0.125 kHz and 16 kHz. Note that correlation strength for tinnitus loudness and delta band power is highest at the fronto-central electrodes contralateral to the tinnitus ear (A), whereas it is highest at the contralateral fronto-temporal electrodes for tinnitus loudness and gamma band power (B). Correlation strengths did not remain significant after FDR correction (FDR 0.05).

Analysis of correlation strength at individual electrode positions revealed differential distribution patterns between oscillatory brain activity and the tinnitus loudness 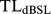 in patients with unilateral tinnitus (Fig. 3). For this analysis electrode positions of left and right hemisphere were mirrored to the contralateral hemisphere in patients with right-sided tinnitus. Using the loudness measure 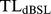 and controlling for age, GPD, and MHL demonstrated an asymmetric distribution of correlation strength between tinnitus loudness and oscillatory band power. Relatively high correlations were observed in the delta (Fig. 3A) and gamma (Fig. 3B) band at frontal electrode positions contralateral to the tinnitus ear. However, none of the correlations remained significant after FDR correction (FDR 0.05).

On the contrary, the loudness measure 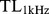, derived from matching the amplitude of a 1 kHz pure tone to the tinnitus loudness, showed no significant correlation with band power averaged over all electrodes in any frequency range. This did not change when controlling for age, GPD, and MHL (Table 3 and 5). Likewise correlating the loudness measure 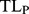, which was derived by adjusting the pure tone corresponding to the major peak in the psychoacoustic tinnitus spectrum to the perceived tinnitus loudness [32], with band power averaged over all electrodes did not show any significant correlation in this analysis. The same was true when 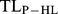 was used instead of 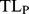.

#### Minimum masking level (MML)

**Figure 4:**
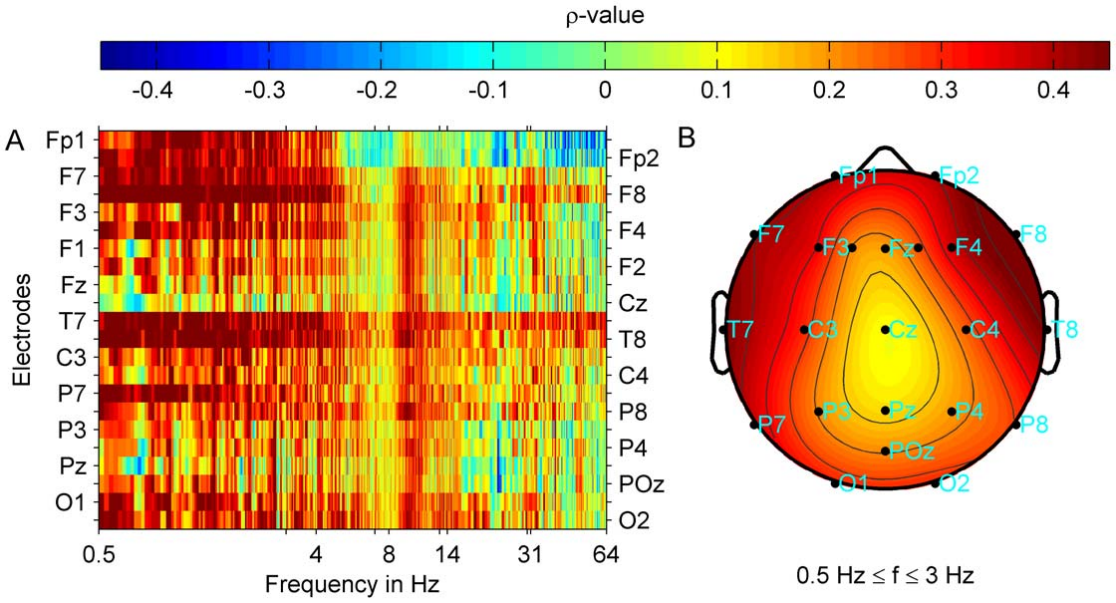
Correlation strength between MML and oscillatory band power. Group average for all tinnitus patients is shown. Power spectra were interpolated with a resolution of 40 points per 1 Hz. Correlations were controlled for age, global psychological distress (GPD) and mean hearing loss (MHL) between 0.125 kHz and 16 kHz. (A) Correlation strength (Spearman's 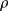) at each electrode and frequency point is shown. Plot (B) shows the correlation map with averaged correlation strength (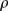) topographies between MML and delta oscillatory power. After FDR correction, correlations at the F8 and T8 electrode position remained significant (FDR 0.05: 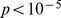).

Increase in delta band power averaged over all electrodes correlated significantly with increasing MML (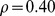, 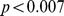). This correlation remained highly significant when controlling for age, GPD, and MHL (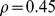, 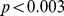), or when controlling for 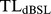 (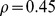, 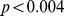), or tinnitus-related distress (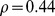, 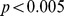) in addition (Table 3 and 5). A detailed analysis (Fig. 4) localized significant correlations at the right fronto-temporal F8 and T8 electrode positions (FDR 0.05: 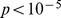).

When subtracting MHL from MML, correlations became significant for all but the theta frequency band, but remained significant only for the delta band when controlling for age, GPD, and MHL (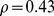, 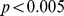) (Table 3, 4, and 5).

#### Tinnitus-related distress and psychometric parameters

**Figure 5:**
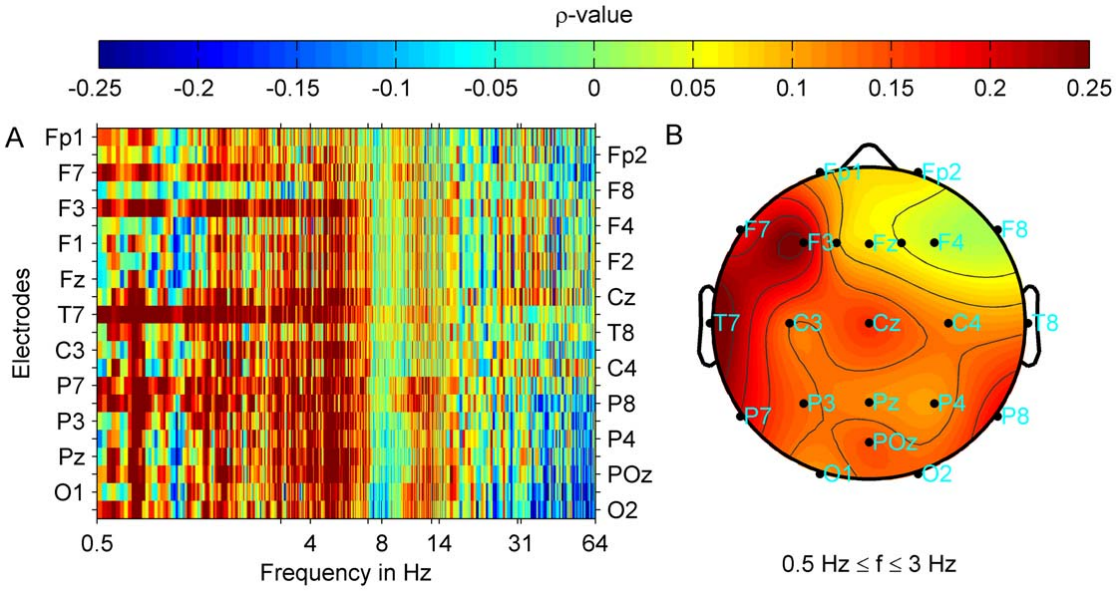
Correlation strength between tinnitus-related distress and oscillatory band power. Group average for all tinnitus patients is shown. Power spectra were interpolated with a resolution of 40 points per 1 Hz. (A) Correlation strength (Spearman's 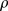) at each electrode and frequency point is shown. Plot (B) shows the correlation map with averaged correlation strength (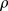) topographies between tinnitus-related distress and delta band power. Irrespective of tinnitus laterality, correlation strength is most pronounced at frontal and temporal locations of the left hemisphere. After FDR correction (FDR 0.05) correlations did not remain significant.

An ANOVA revealed significant differences in theta band power (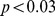) between patients with low and high tinnitus-related distress when averaging power over all electrodes with more theta band power present in the highly distressed patients. A subsequent correlation analysis of band power averaged over all electrodes and tinnitus-related distress showed a significant correlation in the delta band (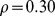, 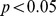), which did not reach significance anymore when controlled for GPD (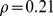, 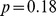). Similarly, correlations between tinnitus-related distress and power in the delta band averaged over all electrodes did not reach significance when controlling for either the depressivity (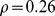, 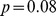), somatization (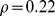, 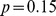), or the anxiety (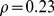, 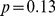) symptom scale of the SCL-90-R, or when controlling for the tinnitus loudness 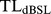 (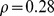, 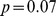). Results of the correlation analyses are given in Tables 3 and 5. When analyzing correlations of tinnitus-related distress and band power at individual electrodes (Fig. 5) correlation strength was highest at frontal and temporal parts of the left hemisphere.

General psychological distress scores (GSI, PSDI, and PST) did not correlate significantly with band power averaged over all electrodes in any frequency band. Likewise, none of the correlations between oscillatory band power and the depression, anxiety or somatization symptom scale scores reached significance (Table 5).

## Discussion

Results of the present study support hypothesis (1) that increasing tinnitus loudness is associated with increasing gamma oscillatory power. This was found to be the case for the tinnitus loudness estimate derived by adjusting the reconstructed tinnitus sound (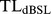) to the perceived tinnitus loudness, but not for the psychoacoustically determined tinnitus loudness estimates determined with other types of sound. In addition, delta band power did significantly increase with the 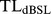 tinnitus loudness estimate. Moreover, increases of loudness-associated gamma and delta activity were localized at the frontal electrodes contralateral to the tinnitus ear for unilateral tinnitus.

Hypothesis (2) that tinnitus loudness and tinnitus-related distress are associated with distinct brain activity patterns was also corroborated by the results of the present study. Tinnitus-related distress does not correlate with gamma band power, and significantly correlates with delta band power at locations which differ from those that correlate with tinnitus loudness. Beyond that high-distress tinnitus was associated with significantly higher theta band power compared to low-distress tinnitus.

The exploratory analysis (3) and (4) revealed that gamma band power decreases with increasing MHL, and that increasing MML correlates with increasing delta band power. In contrast, psychological symptoms such as depressivity and anxiety did not significantly correlate with oscillatory band power.

### Tinnitus loudness and tinnitus-related distress

Recording of spontaneous brain activity revealed increases in oscillatory power in the gamma and delta band related to psychoacoustically determined tinnitus loudness. This increase was significant only when determining tinnitus loudness with the reconstructed tinnitus sound (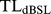), and when controlling for hearing loss, because MHL shows an inverse correlation with gamma band power. In contrast, increases of tinnitus-related distress assessed with the TQ correlated with increases of activity in the delta band only, and highly distressed tinnitus patients exhibited higher theta band power compared to mildly distressed ones. These findings support the distinction between tinnitus loudness and tinnitus-related distress as partly separate aspects of the tinnitus syndrome which was suggested earlier based on questionnaire studies in large tinnitus populations [6], [26]. Most importantly, the present findings extent this distinction to physiological differences suggesting that tinnitus loudness and tinnitus-related distress are related to different pathophysiological mechanisms. A distinction between physiological mechanisms related to tinnitus loudness and distress, respectively, is in line with the results reported by Leaver et al. [7]. These authors observed that neural systems associated with chronic tinnitus differ from those involved in aversive or distressed reactions to the tinnitus. Such a distinction was initially proposed by Jastreboff [44], and it is suggested by the findings of Schlee et al. [27]. As the mean tinnitus-related distress level was rather low in the present study (see Table 1), it is not surprising that the correlation between delta oscillatory power and tinnitus-related distress attains only marginal significance and at this point has to be treated with caution.

In the past, the majority of EEG studies focused on differences between tinnitus and non-tinnitus subjects and did not account for psychological comorbidities although many tinnitus patients suffer from comorbid depressivity or anxiety [45], [46], which themselves might cause changes in oscillatory brain activity [47], [48]. We tested this in the present study and were unable to demonstrate an association between scores in the depressivity, somatization, or anxiety scales of the SCL-90-R questionnaire and band power. This might be due to the circumstance, however, that mean SCL-90-R scores were rather low even though depressivity and anxiety scores were significantly elevated in the present patient group compared to the control group.

Taken together, the results suggest that the tinnitus syndrome can at least be sub-classified into an intensity/loudness category which represents the strength of the tinnitus-related signal, and into a tinnitus-related distress category. In the following, these two aspects will be discussed separately.

### Tinnitus loudness

Tinnitus loudness estimates largely depend on the type of measurement used and it is a matter of controversy which loudness measure represents a valuable estimate. Tinnitus loudness can be assessed in a psychophysical matching procedure in which the loudness of an external auditory signal is matched to the perceived tinnitus loudness [33]. Alternatively, tinnitus loudness can be determined subjectively by ratings on visual analogue scales (VAS) [25]. Fowler [49], [50] report that for most tinnitus patients the psychophysically determined tinnitus loudness was only a few dB above threshold, a statement which was frequently confirmed in subsequent studies [24]. This often contrasts to the high tinnitus loudness that is reported by the patients or that is found with VAS ratings. Fowler called this “the illusion of loudness” [49], [50]. Interestingly in that respect, VAS loudness ratings typically correlate with tinnitus-related distress [6], whereas the psychophysically determined tinnitus loudness does not [24], [33]. Tyler and Conrad-Armes [33] suggested that the discrepancy between psychophysically determined and subjectively experienced tinnitus loudness can be resolved by calculating the psychophysical loudness estimate in the sone scale (see “Psychoacoustic measurements”).

Alternatively, it is possible that the discrepancy between objective and subjective tinnitus loudness estimates originates from recruitment. If a pure tone in a frequency range with significant hearing loss – which is common for the tinnitus frequencies – is used for matching, recruitment phenomena may lead to substantial divergence from the tinnitus loudness estimate derived from matching with a pure tone that corresponds to a frequency region without major hearing loss [33]. An additional factor that influences the perceived loudness of an external sound is its frequency composition since loudness-intensity-functions differ between complex sounds and pure tones [51]. Because the tinnitus spectrum is often complex it appears likely, that the loudness-intensity characteristic of a tinnitus resembles that of a complex sound rather than that of a pure tone.

In the present study, a complex sound, the reconstructed tinnitus spectrum was used for loudness matching in addition to the pure tone corresponding to the major peak of the tinnitus spectrum, and to a 1 kHz pure tone. Because of the reasons outlined above, matching to the complex tinnitus spectrum was expected to achieve better loudness estimates. Logically consistent, only the loudness estimate (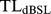) derived from matching with the tinnitus spectrum exhibited a significant correlation with gamma oscillatory activity and at many electrode positions also with delta band power. Correlative strength was not improved by converting this loudness measure to the sone scale (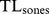). In contrast, tinnitus loudness determined by matching with the pure tone that corresponded to the major peak of the tinnitus spectrum did not show a significant correlation with any frequency band, nor did loudness derived by matching with a 1 kHz pure tone (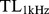). Taken together, tinnitus loudness determined by matching with the complex reconstructed tinnitus spectrum may represent a better loudness estimate than those derived from the commonly used pure tone matching procedures and therefore is recommended for the psychoacoustic determination of tinnitus loudness.

Significant correlations between the tinnitus loudness estimate measured with 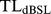 and brain oscillatory activity were seen in the gamma and the delta band. It has repeatedly been shown that gamma band activity (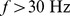) is elevated in tinnitus subjects compared to controls [13]–[15], [18], [20], [52], and similar to the present study previous studies reported elevated gamma oscillations in the hemisphere contralateral to the tinnitus ear for unilateral tinnitus [14], [15], [52] but see [53].

In addition to increases in spontaneous gamma oscillatory activity, increases in delta activity correlated with the 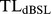 tinnitus loudness measure. This finding is in line with previous MEG studies that reported enhanced delta activity in tinnitus subjects with hearing impairment [11], [12], [54]. Following the thalamocortical dysrhythmia hypothesis originally proposed by Llinas et al. [10], slow wave activity in the delta and theta frequency range is a consequence of input deafferentation, while gamma activity is seen as the tinnitus correlate. Delta activity in tinnitus patients was attenuated by masking [54], as well as during residual inhibition [55], and auditory cortex could be pinpointed as its source [54]. Adjamian et al. [54] did not see a significant correlation between tinnitus loudness and delta band power, which the authors explained by the fact that they correlated subjective loudness rating on a VAS to MEG activity. In contrast, psychoacoustic loudness rating to the reconstructed tinnitus spectrum 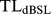 put forth a significant correlation between tinnitus loudness and delta oscillatory activity. Adjamian et al. [54] speculate that increased slow wave activity during wakening may represent synchronized slowing of activity in large populations of neurons with altered thalamic input due to neural deprivation.

Oscillatory activity in the gamma range is furthermore inversely correlated to hearing impairment. This association has not been reported before, and may be owed to the fact that in contrast to other studies (e.g. [13]) hearing impairment was determined for a wider frequency spectrum in the present study which in particular included the high frequency spectrum. In the rodent auditory system, gamma oscillations occur spontaneously and they remain after lesioning the auditory thalamus. This and intracortical recordings suggest that the observed gamma oscillations are generated intrinsically in auditory cortex [56]. Also in a rodent, it was found that age-related hearing impairment is associated with changes in central processing in addition to cochlear impairments [57]. Reduced gamma oscillatory activity during absence of auditory stimulation in a sound-proof environment as seen in the present recordings might therefore be an indicator of reduced auditory cortical functioning in the tinnitus group. In the control group with less hearing impairment between 2 kHz and 10 kHz, no correlation for MHL and gamma band power could be detected.

Although distinct, because it is generated within the auditory system, tinnitus is an auditory percept. Therefore, processing of this signal should in some aspects resemble the processing of external sounds. External sounds evoke event related potentials (ERP), and gamma oscillations are a component of ERP occurring about 100 ms and 300 ms after sound onset in cat hippocampus, reticular formation and cortex [58]. Gamma oscillations have been associated with attention [59], [60] and with emotional content of the sound [61]. This suggests that tinnitus-associated gamma oscillations are influenced by attention and emotion through top-down mechanisms. It is therefore possible that gamma oscillations in tinnitus patients, which were shown to be related to psychophysically determined tinnitus loudness in the present study and to VAS-evaluated tinnitus loudness previously [14], represent activity that is already modified by top-down influences on a primary tinnitus-related signal. Tinnitus loudness obtained by VAS rating shows higher correlations with tinnitus distress than the psychophysiologically determined tinnitus loudness [6]. This in turn might explain the higher correlation between gamma activity and VAS-determined tinnitus loudness [14] as compared to the psychophysiological tinnitus loudness derived by matching with the reconstructed tinnitus spectrum.

In addition, the tinnitus loudness 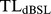 showed significant correlations with delta power at individual electrode positions. In addition to the suggestion that this may be related to auditory deafferentation [54], this can be seen as an expression of the circumstance that tinnitus loudness and tinnitus-related distress are only partially separate aspects of the tinnitus with louder tinnitus usually being associated with more distress (see [6] and below).

In summary, whereas both enhanced gamma and delta activity in the (contralateral) auditory cortex (AC) are associated with tinnitus loudness, only gamma activity is seen as a correlate of the tinnitus percept, and it may be related to attention directed towards and emotions generated by this percept.

### Tinnitus-related distress

Increases of tinnitus-related distress correlate with increases of power in the delta band. This correlation loses significance, however, when controlled for general psychological distress (GPD). GPD does not exhibit a significant correlation with delta band activity on its own, which might be due to the circumstance that mean SCL-90-R scores were rather low even though depressivity and anxiety scores were significantly elevated in the present patient group. Besides that, content overlap between the TQ and SCL-90-R questionnaires may obscure the association between delta band activity and tinnitus-related distress when controlling for GPD. In addition, the association between tinnitus loudness and delta band power might have obscured the association between tinnitus-related distress and delta power in the global analysis, although according to the electrode specific analysis it has its maximum in the right hemisphere whereas correlation strength between tinnitus-related distress and delta band activity peaks in the left hemisphere.

MEG studies also found enhanced delta band power in tinnitus patients compared to controls [10]–[12], which along with Llinas et al. [10] was interpreted as the result of sensory deprivation. An alternative interpretation is suggested by the observation of increased delta activity in depressed elderly patients [48], since tinnitus patients are typically of older age and often express enhanced depressivity (e.g. [6]). Moreover, the P300 auditory evoked response correlates positively with delta EEG power and can be enhanced by emotionally relevant salient stimuli. Salience of stimuli in turn appears to be controlled by dopamine release in nucleus accumbens (NAc). Interestingly, in animals delta oscillations correlate with membrane potential changes in NAc [62]–[64], and D1 agonists of the neurotransmitter dopamine which plays a major role in NAc are known to reduce delta activity [65], [66]. In light of the role of NAc in the tinnitus model put forward by Rauschecker et al. [67], it is tentative to speculate that the association between increases of delta activity, tinnitus-related distress, and to a lesser extent tinnitus loudness is related to dopaminergic activity.

Tinnitus patients with high tinnitus-related distress exhibit higher theta oscillatory power than patients with low tinnitus-related distress. Depressiveness itself is associated with enhanced activity in the theta band [47], [48]. Moreover, hippocampal theta oscillations were shown to strongly associate with anxiety levels in different animal species during various experimental conditions [68]–[71], and they are inhibited by several anxiolytics [71]–[75]. This might explain the higher theta activity in the highly distressed tinnitus patients, while lack of significant correlations between theta power and depressivity as well as anxiety scores of the SCL-90-R may be attributed to their low average level in the present patient population. Whereas gamma oscillations are generated by local circuits, theta oscillations involve larger systems. Slow theta oscillations are generated in a number of brain structures including the hippocampus and parts of the limbic system [76]. They depend on cholinergic input from the medial septum (hippocampal theta) or the basal forebrain (neocortical theta) and are thought to play a role in top-down processing. Theta phase modulation has been implicated in memory retrieval (working memory) and attention [77]. Simultaneous recordings from hippocampus and medial frontal cortex in freely behaving rats indicate that spikes in frontal areas are often phase-locked to the hippocampal theta rhythm, and gamma oscillations generated locally in the neocortex were entrained by this theta rhythm [77]. Furthermore, intracranial recordings in various species and in human epilepsy patients suggest that gamma-theta coupling may contribute to learning and memory formation [77], during which gamma synchrony often couples to the phase of delta or theta oscillations [78]. Therefore, coupling of low frequency and gamma oscillations in tinnitus patients appears likely and it may represent the interaction of the limbic and frontal cortical systems with AC. Enhanced oscillatory brain activity in the gamma range was found to be associated with tinnitus-related distress in some studies [27], [79]. An association that could not be substantiated in the present study, however.

Similarly, the reports on a correlation between tinnitus-related distress with alpha and beta band activity [27], [28], [79] are not supported by the present study. In humans a multitude of factors that are largely independent of tinnitus and are difficult to control for might account for these differences. For example, hunger after overnight fasting is known to influence delta activity [80]. Other reasons could be related to patient selection. This emphasizes the need for a standardization of EEG experiments that investigate tinnitus to allow comparison between studies.

### Minimum masking level

Minimum masking level (MML) represents the lowest level at which external sounds completely mask the tinnitus percept. Maskability by environmental sound provides the patient with an important, if not the only tool to influence his tinnitus percept, therefore it is of utmost clinical importance to understand the underlying mechanism [81]. Furthermore MML has been suggested as a measure for treatment outcome [82]. Mechanisms that account for the observed variance in MML between tinnitus patients are largely unknown, but MML are expected to be associated with tinnitus-related distress [8], and also with the perceived tinnitus loudness. In the present study MML was solely but highly significantly related to delta oscillatory power, and the difference between MML and MHL increased with increasing delta power. This points to an association of MML with tinnitus loudness as well as with tinnitus-related distress. We did not find a significant correlation for MML (or the difference of MML and MHL) and tinnitus-related distress, or for MML and the tinnitus loudness 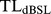 and 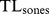, however, whereas in an earlier study with a larger number of severely distressed subjects MML was the only audiologically parameter that showed a significant correlation with tinnitus-related distress [8].

In particular because of its clinical importance, mechanisms underlying tinnitus masking and their association with brain oscillatory activity should be addressed in further studies.

### Conclusion

This is the first report that finds a significant correlation between psychophysically determined tinnitus loudness and brain activity. In line with previous reports the tinnitus percept loudness correlates with gamma and delta oscillatory activity, but only when tinnitus loudness is estimated with a novel type of sound derived by tinnitus reconstruction. We report an easy to apply synthesization paradigm to generate this sound, which comprehensively reflects the spectral complexity of the tinnitus percept and for which patients report extraordinary similarity to their tinnitus. Because this novel tinnitus reconstruction achieves better results than other types of sounds, it is suggested to use it for determining tinnitus loudness in future studies.

The results of the present study also support the clinically motivated distinction between tinnitus loudness and tinnitus-related distress which were shown to associate with distinct patterns of gamma and delta oscillatory brain activity. Additionally, tinnitus-related distress correlates with theta oscillatory activity which is known to be associated with depressivity and anxiety. The increase of delta oscillatory power together with increasing minimum masking level should be investigated in more detail in the future because of its high clinical importance as a tool to control the tinnitus percept.
